# Mainstreaming global mental health: Is there potential to embed psychosocial well‐being impact in all global challenges research?

**DOI:** 10.1111/aphw.12335

**Published:** 2022-01-18

**Authors:** Anna Madill, Netalie Shloim, Brian Brown, Siobhan Hugh‐Jones, Jane Plastow, Diana Setiyawati

**Affiliations:** ^1^ School of Psychology University of Leeds Leeds UK; ^2^ School of Healthcare University of Leeds Leeds UK; ^3^ School of Applied Social Sciences De Montfort University Leicester UK; ^4^ School of English University of Leeds Leeds UK; ^5^ Center for Public Mental Health, Faculty of Psychology Universitas Gadjah Mada Yogyakarta Indonesia

**Keywords:** global development, global mental health, LMIC, mainstreaming, psychosocial well‐being, sustainable development goals

## Abstract

We explore if there is potential to embed psychosocial well‐being impact in global challenges research where the primary aims are not mental health related. We are interested in the use of material practices to deliver impact through routine project activities of working with concrete things together. The UK Research and Innovation (UKRI) gateway to research was searched for information on Global Challenges Research Fund (GCRF) grants from 2015 to May 2020. Analysis shows that only 3 per cent of projects self‐categorise as engaging with mental health. Thirty‐six non‐mental health GCRF grants were purposefully sampled for diversity, and each was coded independently by two researchers for relevant information. Findings suggest that 50–70 per cent of non‐mental health GCRF projects already engage implicitly, but nonstrategically, with psychosocial well‐being impact; opportunities for psychosocial well‐being impact, from most to least frequent, are community mobilisation, community building, skills development, positive sense of self, positive emotions and sociocultural identity; the presence of material practice from most to least frequent is as follows: (i) interactions between or enactments upon people, (ii) written materials or images, and (iii) objects; when a material practice was present, it was usually considered usable as a focus to enhance psychosocial well‐being. Our study provides evidence that there are low hanging fruit opportunities to impact psychosocial well‐being across Sustainable Development Goals (SDGs) through routine project activities.

## INTRODUCTION

Poor mental health can affect anyone, however, particularly vulnerable populations include children, the elderly, people affected by conflicts and disasters, and the disadvantaged (e.g. the poor, women, migrants, minorities, and people with intellectual, cognitive and psychosocial disabilities). Global mental health (GMH) aligns most closely with Sustainable Development Goal (SDG) 3: Good Health and Well‐Being. The extent of the challenge around GMH is huge and requires innovation in how we understand and conduct GMH research, especially now given the human and economic cost of the Covid‐19 pandemic. *Our World in Data* at the University of Oxford reports that, in 2017, 1 billion people had a mental health disorder, placing these among the leading causes of ill health and disability. Poor mental health can lower life expectancy (Lomholt et al., 2019) and disproportionately affects low‐ and middle‐income countries (LMICs) (Patel et al., 2018), where there is also a massive lack of mental health specialists (Bruckner et al., 2011; Herrera‐Ferrá, 2020).

The *Lancet Commission on Global Mental Health and Sustainable Development* (Patel et al., 2018) provides an integration of the empirical evidence and the primary strategic direction driving our research questions (i.e. Questions 1–4 below). The authors conclude that the economic and health case for increased investment in mental health is strong, arguing that mental health is a right and also a means of facilitating sustainable socio‐economic development. The *Lancet Commission* outlines the following priorities: (i) broaden the GMH agenda to the general population; (ii) integrate the global response within other priorities and engage a wide range of stakeholders beyond health; (iii) target social and environmental causes; and (iv) make innovative use of non‐specialists to deliver mental health interventions and to mobilise the voices of those who suffer.

As a way of addressing these priorities, the present study explores if there had been opportunities, in principle, to embed psychosocial well‐being impact in research tackling global challenges across the range of SDGs: in short, to mainstream GMH. Our ambition is to trigger a step change in how the research community thinks about where, how and by whom mental health in LMICs can be impacted. To engage researchers, psychosocial well‐being impact must be deliverable without significant resource implications or overstretching expertise. Hence, we focus on opportunities for contained, yet meaningful, impact that can be undertaken as part of routine project activities. In so doing, we explore the potential for best practice in the application of psychology to the promotion of well‐being by a wide range of stakeholders in the field of GMH and implications for funding bodies who provide strategic direction and resources through financing this work.

The World Health Organization (WHO, 2018) defines mental health as ‘a state of well‐being in which an individual realizes his or her own abilities, can cope with the normal stresses of life, can work productively and is able to make a contribution to his or her community’ (para. 2). To incorporate mental health into sustainable development, De Silva (2015) advises a social determinants framework ‘because it promotes a multi‐sectorial and multi‐disciplinary approach which is the corner stone of good development practice’ (p. 100). This is commensurate with the recommendation of WHO (2020) to consider mental health and psychosocial well‐being adjoining constructs. The terminology of psychosocial well‐being has some benefits for our purpose because it may allow us to identify where diverse projects rub up against mental health through defining broadly what this means; mental health‐related activity in disciplines that do not employ a medical perspective but spans interest at the level of the individual, group, community and region; and routes to impact aligned with SDGs in domains including economic, demographic, environmental, social and cultural (Academy of Medical Sciences [AMS], 2020).

The United Kingdom is an interesting case study because it punches above its weight in the global scientific community, in third position after the United States and China in the 2020 *ScImago Country Rankings*. The Global Challenges Research Fund (GCRF) is a £1.5 billion fund announced by the UK government in late 2015 to support cutting‐edge research that addresses the challenges faced by developing countries. The GCRF therefore plays a key role in determining the kinds of topics that are researched, the methods employed, and relationships cultivated between researchers in wealthier, research‐intensive nations such as the United Kingdom and scientists, practitioners and communities in LMICs. The importance of GMH was recognised in GCRF calls from the Medical Research Council (MRC) in 2017 and 2018 and from the Economic and Social Research Council (ESRC) with the Arts and Humanities Research Council (AHRC) in 2017. All supported multidisciplinary/interdisciplinary working, the MRC focusing on aetiology and epidemiology and the ESRC/AHRC on social and cultural insights.

We are particularly interested to leverage project activities analogous to participatory arts methods because there is a wealth of knowledge that arts methods can benefit psychosocial well‐being in LMICs (e.g. Cooke & Soria‐Dolan, 2019). This literature provides the second strategic direction driving our research questions (i.e. Questions 5 and 6 below). De Silva and Roland (2014) argue that it is important to foster environments promoting well‐being and prevent problems developing through locally inspired, culturally appropriate solutions. Evidence suggests that participatory arts provide a basis for so doing and can impact mental health in terms of prevention and promotion, and management and treatment (Fancourt & Finn, 2019).

Fancourt and Finn (2019) categorise participatory arts for development under five headings: performing arts; visual arts, design and craft; literature; culture; and online/digital. In order to span the gap between the deliberate use of these methods and the vast array of global challenges research in LMICs, we employ the concept of ‘material practices’. This concept captures the ubiquity and diversity of *working with concrete things together* in development‐oriented projects that might, with some creativity, realise analogous, strategically planned psychosocial well‐being benefits. This is supported by de Witte et al. (2021) who argue that a therapeutic factor possibly unique to creative arts is ‘concretization’ defined as ‘changing an abstract content or statement into a tangible form that can be physically perceived, experienced, and related to’ (para. 48). Similarly, material practices in research include the use of *text*, such as writing, diagrams and images (Piquette & Whitehouse, 2013); *objects*, such as artefacts, implements and tangible structures; and interactions between, enactments upon, *people*: all facets of participatory arts in the context of development initiatives. We chose to focus on where research used material practices because they are routine in many kinds of project, are well suited to work in LMICs given their inclusiveness and capacity to reach marginalised groups (Duara et al., 2018), engage people irrespective of education and language, can be inexpensive and integrate local traditions (Boon & Plastow, 2004).

We take inspiration for our work from the *Building Resilience Through Heritage* (BReaTHe) initiative led by Croucher, Evans, Greene and Wilson at the University of Bradford, UK, in partnership with Mercy Corps. As an aspect of public engagement, the team's initial GCRF‐funded research, *Augmenting Jordanian Heritage*, created opportunities through the use of three‐dimensional visualisations and printed models for refugees to handle virtually and manipulate objects representing heritage that had been destroyed or vandalised in recent conflict. Recognising the mental health impact of these activities, the team secured funding for GCRF project *Building Resilience Wellbeing and Cohesion in Displaced Societies Using Digital Heritage*. This second project is a novel interdisciplinary collaboration between archaeology, digital heritage, peace studies and international development. It uses heritage in an innovative way to enhance a sense of place and to explore the role and value of digital heritage in identity, community development and well‐being with conflict‐affected communities. Specifically, it enables people to discuss and use digital heritage for community confidence building, cohesion and psychosocial well‐being. These heritage‐oriented GCRF projects provide evidence of the potential for global researchers to impact mental health, and to learn to do so strategically, while delivering their core, non‐mental health, project aims.

In summary, we explore if there is potential to embed psychosocial well‐being impact in global challenges research where the primary aims are not mental health related. Our approach at this juncture is discovery oriented and descriptive, as opposed to hypothesis testing, given the current dearth of knowledge in this area. We anticipate our work contributing to the development of hypothesis‐testing research in the future. We examine information published by UK Research and Innovation (UKRI) to address our six research questions.

Our first four research questions are driven by the priorities outlined by the *Lancet Commission on Global Mental Health and Sustainable Development* described above. The first two are as follows: (1) Where are GCRF funds allocated? (2) To what extent do GCRF projects self‐categorise as engaging with mental health? This information is important in establishing relevant context. Specifically, given the remit of GCRF to addresses the challenges faced by developing countries, it is important to know the relative position of mental health in terms of research interest and investment. Research Questions 3 and 4 address the potential of non‐mental health GCRF projects to engage psychosocial well‐being impact: (3) To what extent do non‐mental health GCRF projects engage implicitly with mental health (defined broadly as psychosocial well‐being)? (4) What were the opportunities to impact the psychosocial well‐being of target groups had only minor changes been made to the project? Our final two research questions are driven by the literature on participatory arts methods in LMICs: (5) Does the project involve any texts, objects or interactions between or enactments upon people? (6) To what extent might these create a focus to enhance psychosocial well‐being with target groups? Research Questions 3–6 allow us to establish if mental health impact, and potential for mental health impact, is being leveraged as effectively and strategically as possible in global challenges research as represented by the GCRF.

## GCRF CONTEXT: ANALYSIS OF GRANTS BY CLASSIFICATION ON GATEWAY TO RESEARCH

To address these six research questions, we developed a data collection and analysis strategy to interrogate the UKRI gateway to research (GtR: https://gtr.ukri.org/). This is a searchable database allowing analysis of information on publicly funded UK research. GtR was the main source of data for our study and where other sources are used these are specified. GtR includes grants funded by the following organisations, all of which are independent, nondepartmental public bodies of the UK government's Department for Business, Energy and Industrial Strategy: AHRC, Biotechnology and Biological Sciences Research Council (BBSRC), ESRC, Engineering and Physical Sciences Research (EPSRC), MRC, Natural Environment Research Council (NERC), and Science and Technology Facilities Council (STFC). GtR was searched for information on GCRF grants from the start of the programme in late 2015 until the end of May 2020.

### Research Question 1: Where are GCRF funds allocated?

UKRI publishes data on competitive funding awards. Data for 2015–2020 (updated as of 18 November 2020) were found at https://public.tableau.com/app/profile/uk.research.and.innovation.ukri./viz/CompetitiveFundingDecisions2015-16to2019-20/UKRICompetitiveFunding. Table 1 shows that, in terms of the percentage of total GCRF grants until the end of May 2020, the largest single proportion was awarded via the MRC (29%), followed by the AHRC (23%), then closely by the ESRC (22%). This is the same order in which the research councils hit above their weight with respect to number of GCRF grants compared with their total number of grants 2015–2020: MRC (+14%), AHRC (+13.5%) and ESRC (+11%). The lowest number of GCRF grants was awarded via the STFC (3%), the NERC (6%) and the BBSRC (7%). However, in terms of number of GCRF grants compared with total number of grants 2015–2020, it is the EPSRC who awarded the least proportion (−17.5%), followed by the BBSRC (−8.5%) and the NERC (−8%).

**TABLE 1:** Total number of grants awarded by UKRI research councils 2015–2020, total number of GCRF grants awarded and number of GCRF grants mental health related

Research council	No. of grants 2015–2020 (% of total UKRI grants)	No. of GCRF grants 2015 to May 2020 (% of total GCRF)	GCRF above (+%)/below (−%) weight	No. of GCRF grants mental health^a^
AHRC	1461 (9.5%)	269 (23%)	+13.5%	1^b^
BBSRC	2384 (15.5%)	86 (7%)	−8.5%	0
EPSRC	4219 (27.5%)	124 (10%)	−17.5%	1^c^
ESRC	1667 (11.0%)	275 (22%)	+11.0%	7^d^
MRC	2217 (14.5%)	351 (29%)	+14.5%	27
NERC	2076 (14.0%)	79 (6%)	−8.0%	0
STFC	1255 (8.0%)	40 (3%)	−5.0%	0
Total	15279 (100%)	1224 (100%)	—	36
		**% GCRF mental health**		3%

Abbreviations: AHRC, Arts and Humanities Research Council; BBSRC, Biotechnology and Biological Sciences Research Council; EPSRC, Engineering and Physical Sciences Research Council; ESRC, Economic and Social Research Council; GCRF, Global Challenges Research Fund; MRC, Medical Research Council; NERC, Natural Environment Research Council; STFC, Science and Technology Facilities Council; UKRI, UK Research and Innovation.

^a^ Mental health ‘research topic’/MRC health ‘health category’.

^b^ Plus the seven cofunded with, and led by, ESRC.

^c^ The current project.

^d^ Cofunded with AHRC.

Applications to the seven UK research councils are submitted via the online Joint Electronic System (JeS). During the grant submission process, at least one research area must be selected from a list provided indicating relevant subject area(s) or discipline(s). Each research area has subcategories from which more detailed terms can be selected. Applicants are instructed to select terms at the lowest appropriate level to describe the project. Qualifiers that further describe the area of study are grouped by type such as health category, approach, geographic area and theoretical methods. Applicants are instructed to select as many qualifiers as are relevant to enable understanding of the proposal. MRC applications have been largely brought into line with the other research councils. However, within the time frame of the data collection for this article, the relevant application classification is the health category qualifier only (Table 2).

**TABLE 2:** Proposal classifications allowing identification of mental health related grants

Research council	JeS	GtR summary
All except MRC	Research area ‘medical and health interface’	Research subject (not searchable)
	Subcategory ‘mental health’	Research topics (total *N* = 610)
	Qualifier ‘health categories’	Health categories (total *N* = 23) (filter search required)
	Subcategory ‘mental health’	
MRC	Qualifier ‘health categories’	Health categories (total *N* = 23)
	Subcategory ‘mental health’	Research activity (not searchable)

Abbreviations: GtR, gateway to research; JeS, Joint Electronic System; MRC, Medical Research Council.

To establish what has attracted the most funding, research topics with frequency (*f*) ≤25 and, for MRC grants, health categories with *f* ≤ 20 were identified for the total sample of GCRF grants. These frequencies were decided through inspecting the GtR data and determining what would capture popular classifications while not overly narrowing focus. The most frequently used of these are as follows: (i) agricultural systems, (ii) natural resources, environment and rural development, (iii) anthropology and development and, for the MRC, (iv) infection and (v) generic health relevance.

Popular research topics/healthc categories were then clustered inductively into themes as a way of identifying where GCRF funds are allocated. At this stage, five high‐frequency research topics were omitted as too generic to allow thematic clustering for our purposes: African studies (*f* = 42), Asian and Middle Eastern studies (*f* = 30), development studies (*f* = 124), postcolonial studies (*f* = 30), and women's and gender studies (*f* = 26). The outcome of this thematic clustering was very similar to the six named GCRF strategic challenge portfolios, hence, serendipitously, reconstructing the programme structure. This demonstrates the close fit between the GCRF strategic challenge portfolios and funded GCRF research. It was decided therefore to use the portfolio titles to organise the GCRF‐funded topics (Table 3).

**TABLE 3:** High frequency funded research topics and mental health related grants associated with GCRF strategic challenge portfolios

GCRF strategic challenge portfolio	Research topics *f* ≤ 25/MRC health categories *f* ≤ 20	Research topic/MRC health category coclassifications with GCRF mental health grants
Cities and sustainable infrastructure	Social policy and development (*f* = 41) Economic development (*f* = 35) Heritage management (*f* = 30) Development economics (*f* = 25)	**Social policy** (*f* = 3) **Economic development** (*f* = 1) **Development economics** (*f* = 1) Museum and gallery studies (*f* = 1) **Social policy and development** (*f* = 1)
Education	International education and development (*f* = 30) Education (*f* = 26)	Education policy (*f* = 1)
Food systems	Agricultural systems (*f* = 71) Soil science (*f* = 27) Crop science (*f* = 25)	—
Global health		
Non‐MRC	Community art including art and health (*f* = 46) Global health and medicine (*f* = 36)	**Community art including art and health** (*f* = 2) Biomedical neuroscience (*f* = 1) Cognitive psychology (*f* = 1) **Global health and medicine** (*f* = 1) Health psychology (*f* = 1) Medical sociology/sociology if health and illness (*f* = 1)
MRC	Infection (*f* = 120) Generic health relevance (*f* = 73) Mental health (*f* = 27) Reproductive health and childbirth (*f* = 25) Cancer and neoplasms (*f* = 23)	**Generic health relevance** (*f* = 2) **Infection** (*f* = 1) **Reproductive health and childbirth** (*f* = 1)
Resilience to environmental shocks and change	Natural resources, environment and rural development (*f* = 58) Geography and development (*f* = 48) Climate and climate change (*f* = 39) Regional and extreme weather (*f* = 35) Environmental geography (*f* = 26) Geohazards (*f* = 25)	—
Security, protracted conflict, refugee crises and forced displacement	Anthropology and development (*f* = 52) Politics, international relations and development (*f* = 36) Peace studies (*f* = 35) Social anthropology (*f* = 32) Conflict/war studies (*f* = 29) Cultural history (*f* = 27)	Psychology (*f* = 2) Social psychology (*f* = 2) Children and families (*f* = 1) Cultural geography (*f* = 1)

*Note*: Research topic/MRC health category coclassification in bold is also one of the most popular classifications within portfolio.

Abbreviations: GCRF, Global Challenges Research Fund; MRC, Medical Research Council.

### Research Question 2: To what extent do GCRF projects self‐categorise as engaging with mental health?

Although non‐MRC projects can be classified on submission under the ‘mental health’ health category, this is not displayed on the GtR grant summary page. A specific filter search was therefore conducted, which revealed that no non‐MRC GCRF grants were self‐categorised under the ‘mental health’ health category. Table 1 shows that, in total, only 3 per cent (*N* = 36) of GCRF grants were classified by applicants at submission as mental health related. Virtually all of these were via the MRC (*N* = 27), with the rest funded via joint AHRC/ESRC funding (*N* = 7), one via the AHRC alone and one (the present project) via EPSRC hosting of the Global Challenge Cluster programme.

GCRF grants self‐categorised on application as mental health related also self‐categorised with research topics/health categories relevant to four of the six GCRF strategic challenge portfolios, in order of frequency: global health (*f* = 34), cities and sustainable infrastructure (*f* = 7), security, protracted conflict, refugee crises and forced displacement (*f* = 6), and education (*f* = 1) (Table 3). Finally, mental health‐related grants were commensurate with key areas in two GCRF strategic challenge portfolios, in order of frequency: global health (*f* = 7), and cities and sustainable infrastructure (*f* = 6) (Table 3).

### Discussion: Research Questions 1 and 2

Our analysis reveals some interesting patterns in relation to the GCRF and GMH. GCRF funding allocation has been commensurate with its strategic challenge portfolios but under a relatively limited number of key research topics (23 of 610) and key health categories (5 of 23). Mental health is the third most frequently used MRC health category, but by a large gap, and in total only 3 per cent of GCRF grants self‐categorise as mental health related. These findings indicate very little emphasis on GMH in the programme and underscores the urgency of the work undertaken recently by the UK Department for International Development to formalise its position on, and approach to, the integration of mental health across diverse development sectors (https://www.mhinnovation.net/mental-health-and-international-development-launch-dfidfcdo-position-paper-and-voices-field).

Interestingly, mental health aligns best with only two GCRF strategic challenge portfolios: (i) global health and (ii) cities and sustainable infrastructure. Hence, where it has been engaged, GMH has tended to be situated in a biomedical model and/or to be framed within social policy and economic development. Mental health alignment with cities and sustainable infrastructure appears to engage many of the *Lancet Commission* priorities, that is, to broaden the GMH agenda to the general population, integrate the global response within other priorities and engage a wide range of stakeholders beyond health, and to target social and environmental causes. However, engagement is still narrow.

Mental health‐related grants were tangentially aligned with two GCRF strategic challenge portfolios: (i) security, protracted conflict, refugee crises and forced displacement, and (ii) education. With regard to the former, there is a mass of evidence that conflict and forced displacement can create multigenerational trauma and severely compromise the social networks through which communities build and sustain resilience (e.g. Siriwardhana & Stewart, 2013). With regard to the latter, educational organisations can be both the source and site of interventions to enhance the life chances of young people and the vitality of the communities served (Patalay et al., 2017). GMH carries a wide range of meanings (Rajabzadeh et al., 2021), and to establish a foothold in the development sectors associated with these two GCRF portfolios, we may need to convey the potential for mental health impact in a language that resonates better with researchers in these fields, in particular that avoids implicating a narrow biomedical model.

Mental health‐related grants were not aligned with the remaining two GCRF strategic challenge portfolios: (i) food systems and (ii) resilience to environmental shocks and change. This suggests that it may be even more difficult to argue for the relevance of psychosocial well‐being impacts in related fields. However, our coders agreed that 50 per cent of the former and 67 per cent of the latter projects sampled within these GCRF portfolios already engaged implicitly with the mental health of target groups.

## POTENTIAL OF NON‐MENTAL HEALTH GCRF PROJECTS TO EMBED PSYCHOSOCIAL WELL‐BEING IMPACT: ANALYSIS OF A SAMPLE OF GRANTS ON GtR

Having established the broader funding context, we now address our research questions regarding the potential that non‐mental health GCRF projects had, in principle, to embed psychosocial well‐being impact in their work.

### Sampling strategy

**FIGURE 1:**
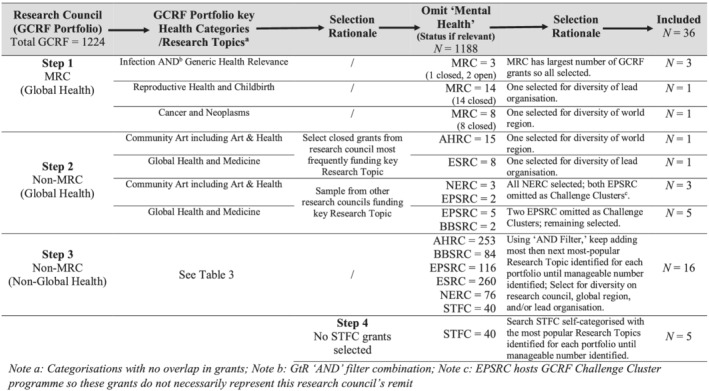
Sampling strategy process from gateway to research (GtR). AHRC, Arts and Humanities Research Council; BBSRC, Biotechnology and Biological Sciences Research Council; EPSRC, Engineering and Physical Sciences Research Council; ESRC, Economic and Social Research Council; GCRF, Global Challenges Research Fund; MRC, Medical Research Council; NERC, Natural Environment Research Council; STFC, Science and Technology Facilities Council

A sample of GCRF grants was required whose primary research aim was not mental health, that is, which were not classified on GtR as ‘mental health’ research topic or health category (MRC) (Table 4). Purposive sampling for diversity was used across research council, GCRF strategic challenge portfolio and world region. We used the WHO world region categorisation: Africa, Americas, Southeast Asia, Europe, Eastern Mediterranean and Western Pacific (https://www.who.int/about/who-we-are/regional-offices). Where possible, closed (total pool *N* = 484) rather than active (total pool *N* = 740) grants were selected for completeness of available GtR information. Differentiation of lead organisation and research category was also sought. Most GCRF grants are research category ‘research grant’ (*N* = 1110) followed by ‘intramural’ (*N* = 64), ‘fellowship’ (*N* = 44) and ‘training grant’ (*N* = 6). A sample of 36 grants was identified in four steps (Figure 1).

**TABLE 4:** Sampled grants by GCRF portfolio, research council, status and region

GCRF strategic challenge portfolio	Research council	Active/closed	Global region
Cities and sustainable infrastructure (*N* = 6)	AHRC	C	SE Asia
	EPSRC	A	Global^a^
	ESRC	A	Africa
			Global
		C	Africa/Americas/SE Asia/Western Pacific^a^
			Eastern Mediterranean/Europe/SE Asia
Education (*N* = 5)	AHRC	A	Americas
			Eastern Mediterranean
			SE Asia
	ESRC	C	Africa
			Americas^b^
Food systems (*N* = 6)	BBSRC	A	Africa^a^
			SE Asia
		C	SE Asia/Western Pacific^a^
	STFC	C	Africa
			Americas^a^
			Eastern Mediterranean/SE Asia/Western Pacific
Global health (*N* = 15)	AHRC	C	Americas/Eastern Mediterranean
	BBSRC	C	Africa^a^
			Africa
	EPSRC	A	Africa^c^
			Americas^c^
			Europe^a^
	ESRC	C	Africa^a^
	MRC	A	Africa^c^ ^,^ ^d^
			Western Pacific^a^
		C	Americas/Western Pacific
			Africa
			SE Asia^c^
	NERC	C	Africa
			Africa/Western Pacific^a^
			SE Asia
Resilience to environmental shocks and change (*N* = 3)	NERC	C	Africa
	STFC	C	Americas
			Global^a^
Security, protracted conflict, refugee crises and forced displacement (*N* = 1)	AHRC	C	Americas

Abbreviations: AHRC, Arts and Humanities Research Council; BBSRC, Biotechnology and Biological Sciences Research Council; EPSRC, Engineering and Physical Sciences Research Council; ESRC, Economic and Social Research Council; GCRF, Global Challenges Research Fund; MRC, Medical Research Council; NERC, Natural Environment Research Council; SE, Southeast; STFC, Science and Technology Facilities Council.

^a^ Both coders agree no implicit engagement with psychosocial well‐being.

^b^ Fellowship.

^c^ Non‐UK lead organisation.

^d^ Intramural.

Table 5 shows that, as planned, our sample contains a higher proportion of closed grants (64%) than in the available pool (40%). EPSRC grants are under‐represented by 16.5 per cent, African‐based projects by 19 per cent, and GCRF strategic challenge portfolios (i) security, protracted conflict, refugee crises and forced displacement and (ii) resilience to environmental shocks and change by 22 per cent and 19 per cent, respectively. On the other hand, GCRF strategic challenge portfolio global health is over‐represented in our sample by 31 per cent. This is a result of purposive sampling for diversity in order to explore possibilities for embedding psychosocial well‐being impact across different types of project.

**TABLE 5:** Sampled GCRF grants compared with total GCRF grant by status, research council, region and GCRF portfolio

Dimension	GCRF total	Sample	Sample above (+%)/below (−%) GCRF
Status			
Active	740 (60%)	13 (36%)	−24%
Closed	484 (40%)	23 (64%)	+24%
Research council			
AHRC	1461 (9.5%)	6 (17%)	+7.5%
BBSRC	2384 (15.5%)	5 (14%)	−1.5%
EPSRC	4219 (27.5%)	4 (11%)	−16.5%
ESRC	1667 (11.0%)	7 (19%)	+8.0%
MRC	2217 (14.5%)	5 (14%)	−0.5%
NERC	2076 (14.0%)	4 (11%)	−3.0%
STFC	1255 (8.0%)	5 (14%)	+6%
Global region			
Africa	515^a^ (49%)	14^b^ (30%)	−19%
Americas	137 (13%)	9 (19%)	+6%
Southeast Asia	170 (16%)	9 (19%)	+3%
Europe	28 (3%)	2 (4%)	+1%
Eastern Mediterranean	82 (8%)	4 (9%)	+1%
Western Pacific	119 (11%)	6 (13%)	+2%
Global	—	3 (6%)	—
GCRF strategic challenge portfolio			
Cities and sustainable infrastructure	131 (16%)	6 (17%)	+1%
Education	56 (7%)	5 (14%)	+7%
Food systems	123 (15%)	6 (17%)	+2%
Global health	82 (10%)	15 (41%)	+31%
Resilience to environmental shocks and change	231 (27%)	3 (8%)	−19%
Security, protracted conflict, refugee crises and forced displacement	211 (25%)	1 (3%)	−22%

Abbreviations: AHRC, Arts and Humanities Research Council; BBSRC, Biotechnology and Biological Sciences Research Council; EPSRC, Engineering and Physical Sciences Research Council; ESRC, Economic and Social Research Council; GCRF, Global Challenges Research Fund; MRC, Medical Research Council; NERC, Natural Environment Research Council; STFC, Science and Technology Facilities Council.

^a^ https://public.tableau.com/app/profile/research.councils.uk/viz/RCUKGCRFFundedProjects/RCUKGCRFAwardedProjects as of 27 April 2018, accessed 6 June 2021.

^b^ All regions mentioned in Table 4 counted independently (total *N* = 47).

### Data extraction

Data consist of all the grant information available on GtR. Summary information for each grant has the following typical content: overview—title, funded value, funded period, funder, project status, project category, project reference, principal investigator, research subject (categories), research topic (categories), research programme (i.e. GCRF), abstract, technical summary and planned impact; organisations—lead organisation and collaborators; people—principal investigator, coinvestigator(s), cofunder(s) and project partner(s); publications (if available); and outcomes (if available)—artistic and creative products, key findings, impact summary, research tools and methods, collaboration, software and technical products, and engagement activities. A number of steps were taken to develop a template to guide and record data extraction of information available on GtR.

Three sources were inspected for key types of mental health impact relevant to LMIC contexts (AMS, 2020; Fancourt & Finn, 2019; WHO, 2018). There emerged a high‐order clustering, each with three facets, under (i) psychological well‐being impact (i.e. related to personal development) and (ii) social well‐being impact (i.e. related to enhanced connections and community influence) (Table 6). Having created a condensed list of psychological and social types of mental health impact relevant to LMICs, the first author designed an initial data extraction template. The template was piloted on five grants in our sample by the first and second authors and revised through several iterations. The template was then piloted on two of these grants with the third, fourth and fifth authors and revised again. The final template notes the coder's name, grant title and grant GtR link. The coder is asked to identify: Which target groups of this project could be the focus of psychosocial well‐being impacts? They were then asked to address the following questions with regard to these target groups: What aspects of this project engage implicitly with the psychosocial well‐being of target groups? What were the (missed) opportunities to impact the psychosocial well‐being of target groups had only minor changes been made to the project? Does the project involve any written materials or images? Does the project involve any objects (i.e. artefacts, implements and tangible structures), building or making something? Does the project involve any interactions between or enactments upon people? And, for the latter three questions, how might these create a focus to enhance psychosocial well‐being with target groups?

**TABLE 6:** Psychological and social types of mental health impact relevant to low‐ and middle‐income countries

Psychological well‐being	Social well‐being
Promote positive sense of self (e.g. self‐esteem, self‐acceptance, self‐expression, self‐efficacy, confidence and independence) Promote positive emotions, safe expression and regulation of challenging emotions, coping strategies and help‐seeking Facilitate skills development (e.g. life skills, relationship skills, parenting skills and healthy lifestyle skills)	Facilitate community building (e.g. social support, mutual engagement, group belonging, trust and solidarity) Preserve sociocultural identity (e.g. religious, ethnic, cultural and heritage) Support community mobilisation (e.g. awareness raising, socio‐economic empowerment, reduction of stigma, discrimination and social exclusion)

### Analytical procedures

The sampled grants were distributed randomly across a team of 14 academics such that the data extraction template was completed for each of the 36 grants independently by two coders. All but one of these academics has been involved in GCRF grants as a principal or coinvestigator. Their disciplinary backgrounds span: African studies; archaeology and heritage; community, health, and mental health psychology; counselling and psychotherapy; English and theatre; global health policy; international development; participatory arts; nursing; and social anthropology. Coder agreement is presented as a percentage given that the assessment task is relatively interpretative and was to explore possibilities rather than to determine certainties. Hence, following Glen (2016), we set an acceptable level of agreement at 60 per cent and strong at 70 per cent.

### Research Question 3: To what extent do non‐mental health GCRF projects engage implicitly with mental health (defined broadly as psychosocial well‐being)?

Information in the completed data extraction templates to the question ‘To what extent do non‐mental health GCRF projects engage implicitly with mental health (defined broadly as psychosocial well‐being)?’ was tabulated into the form ‘no engagement’ and ‘engagement’. Coding agreement was strong at 84 per cent. For 11 grants (31%), both coders agreed that there was no implicit engagement with psychosocial well‐being impact (Table 4: cities *N* = 2; food systems *N* = 3; global health *N* = 5; and resilience *N* = 1). For 19 grants (53%), both coders agreed that there was implicit engagement with psychosocial well‐being impact, and for six grants (17%), one of the two coders considered there to be implicit engagement. Hence, at least one coder considered there to be implicit engagement with psychosocial well‐being impact in 69 per cent of the sampled grants.

### Research Question 4: What were the opportunities to impact the psychosocial well‐being of target groups had only minor changes been made to the project?

Agreement between the two coders of each project was strong on opportunities for psychosocial well‐being impact on the three social types: support community mobilisation (92%), facilitate community building (83%) and preserve sociocultural identity (70%). Coding agreement was acceptable on opportunities for psychosocial well‐being impact on two of the psychological types: promote positive emotions (67%) and facilitate skills development (61%), and unreliable on positive sense of self (58%) (Figure S1). Agreed perceived opportunities for psychosocial well‐being impact, from most to least frequent type are as follows: support community mobilisation (92%), facilitate community building (75%), facilitate skills development (61%), promote positive sense of self (50%), promote positive emotions (44%) and preserve sociocultural identity (42%). All projects were considered to have an opportunity to support community mobilisation and/or to facilitate skills development by at least one coder (Figure S2). Each project was agreed by the coders to have at least one opportunity to enhance psychosocial well‐being (range 1–6, mode = 2, mean = 4) (Figure S3). The two coders for each project did not necessarily agree which types of opportunity are present, meaning that this information is the most generously indicative of potential for psychosocial well‐being impact.

### Research Question 5: Does the project involve any (i) written materials or images (texts); (ii) objects (i.e. artefacts, implements and tangible structures), building or making something; and (iii) interactions between or enactments upon people?

Coding agreement was strong for the presence of people (75%) and objects (75%) and acceptable for texts (61%). Relative lack of confidence in identifying texts was reflected in that one of the coders of an additional 17 per cent indicated that they were ‘not sure’, compared with an additional 14 per cent for objects and only an additional 8 per cent for people (Figure S4). Agreed presence of type of material practice from most to least frequent is as follows: people (72%), texts (47%) and objects (25%). When considered present by at least one coder, this rises to 97, 75 and 39 per cent, respectively (Figure S5). Coders agreed that: two projects had no material practices present; three had one type; 20 had two types; and 11 had three types (range = 0–3, mode = 2, mean = 2). The two coders for each project did not necessarily agree which types are present, meaning that this information is the most generously indicative (Figure S6). However, each project was considered to involve at least one type of material practice by at least one coder.

### Research Question 6: To what extent might these create a focus to enhance psychosocial well‐being with target groups?

From most to least frequent, agreed usability of type of material practice to create a focus to enhance psychosocial well‐being is as follows: people (64%), texts (36%) and objects (14%). When considered usable by at least one coder, this rises to 92, 69 and 22 per cent, respectively (Figure S7). Coders agreed that: four projects contained no material practices that could have been used to enhance psychosocial well‐being: 16 contained one type that could have been used in this way; 13 contained two types; and three contained all three types (range = 0‐3, mode = 1, mean = 1.4) (Figure S8).

### Discussion: Research Questions 4–6

In total, our analysis suggests that between 50 and 70 per cent of non‐mental health GCRF projects engage implicitly with psychosocial well‐being impact and each to have had at least one opportunity, in principle, to enhance the psychosocial well‐being of target groups. This supports that there is potential to embed psychosocial well‐being impact in global challenges research where the primary aims are not mental health related. Much of this work is already being done, likely unrecorded and, because implicit, is unlikely to be developed strategically for sustainable impact. Importantly, although mental health‐related grants were not aligned with two GCRF strategic challenge portfolios, i.e. (i) food systems and (ii) resilience to environmental shocks and change, coders agreed that 50 per cent of the former and 67 per cent of the latter projects sampled within these portfolios already engaged implicitly with the mental health of target groups.

Opportunities for social well‐being impact (i.e. related to enhanced connections and community influence) were more reliably identifiable than opportunities for psychological well‐being impact (i.e. related to personal development). This could have been due to the restricted information available on GtR. On the other hand, it may have been easier to identify social well‐being impacts because the GCRF programme requires strong LMIC partnerships and collaboration. This is reflected in our finding that the social well‐being impacts of community mobilisation and of community building were the most frequently identified opportunities. Moreover, all projects were considered to have an opportunity to support community mobilisation and/or to facilitate skills development by at least one coder. More work is required to understand how opportunities for psychosocial well‐being impact can be identified in non‐mental health‐focused projects, particularly psychological impacts.

To leverage mental health impact, we chose to explore the potential of participatory arts, conceptualised broadly as material practices, because we anticipated that a wide variety of GCRF projects presented opportunities to work with concrete things together. Importantly, when a material practice was present, it was usually considered usable as a focus to enhance psychosocial well‐being, especially when a project included interactions between people.

There is a growing literature emphasising the inherent value of building social relationships, social networks and working collaboratively with people in developing countries (Perkins et al., 2015). Interventions to increase, what we might call, social capital in LMICs and associated psychosocial well‐being impacts are flourishing: ‘community engagement and educative programs, cognitive processing therapy and sociotherapy for trauma survivors, and neighbourhood projects’ (Flores et al., 2018, p. 107). However, lack of agreed definition of social capital can create problems for evidencing impact and the longevity of positive outcomes has yet to be established. Arguably, the three social impacts we identify as aspects of psychosocial well‐being relevant to LMICs (Table 6) usefully combine the social *cohesion* and the social *network* lens on social capital (Ehsan et al., 2019) and, hence, could provide a pragmatic way of mapping this terrain, at least for our purposes.

The psychotherapy literature also endorses that good‐quality relationships are central to promoting mental health. For example, research supports the effectiveness of Rogers' three core conditions for therapeutic change: that the counsellor is genuine, conveys acceptance and demonstrates empathy (Kirschenbaum & Jourdan, 2005). Importantly, for low‐resource settings, these are also non‐technical skills implicit to routine, collaborative working with partners and participants. Moreover, therapeutic alliance research reveals that the relationship between client and therapist is key to change and has three components: tasks, goals and bonds (Falkenström et al., 2016). These components are also implicit to collaborative working, with material practices potentially providing a focus—the tasks and goals—around which bonds can develop. Moreover, the potential has been demonstrated for talking therapies to be delivered by lay people in the aftermath of humanitarian crises in LMICs with the interventions found generally to be ‘acceptable, appropriate and feasible to implement, with good fidelity to manualised therapies’ (Ryan et al., 2021, p. 1). We can draw confidence from this work that global challenges researchers, similarly, might be supported to embed psychosocial well‐being impacts within the material practices and collaborative process with which they are already extremely familiar and skilful, the type of material practice less important than the collaborative potential held (Colucci & Bhui, 2015).

## CASE STUDIES

We now present three short case studies in which the quantitative data are illustrated within the context of actual grants in our sample, informed by the qualitative notes in the data extraction template by the relevant two coders and, for Cases 1 and 3, a research interview with the principal investigator/fellow (Ethics Committee, School of Psychology, University of Leeds: PSYC‐26, 21 April 2020; the case study interviewees have given written consent to be identifiable). The three case studies were selected carefully as positive examples of opportunity to embed psychosocial well‐being impact in non‐mental health projects in principle, and the two for which interviews are available were selected from a corpus of 26 interviews conducted in the second phase of our work (Madill et al., in submission). Closed projects were selected for completeness of information on GtR and then sampled for diversity (Table 7). The three selected projects were held at different UK universities and were coded by five different team members, two per project.

**TABLE 7:** Summary information on case study projects

Case	Research council/project category	GCRF strategic challenge portfolio	World region	Both relevant coders agree are present		
				Psychological well‐being opportunity	Social well‐being opportunity	Usable material practice
1	ESRC Fellowship	Education	Americas	All	All	Text
2	AHRC Research grant	Cities and sustainable infrastructure	SE Asia	Promote positive sense of self	Facilitate community building Support community mobilisation	Objects People
3	NERC Research grant	Resilience to environmental shocks and change	Africa	None	All	People

Abbreviations: AHRC, Arts and Humanities Research Council; ESRC, Economic and Social Research Council; GCRF, Global Challenges Research Fund; NERC, Natural Environment Research Council; SE, Southeast.

### Case 1: Levelling the playing field: Assessing through equity the quality of Chilean schools

Our findings demonstrate that mental health‐related grants were tangentially aligned with two GCRF strategic challenge portfolios: (i) security, protracted conflict, refugee crises and forced displacement, and (ii) education. With regard to the latter, Case Study 1 illustrates how educational organisations can be both the source and site of interventions to enhance the life chances of young people and the vitality of the communities served.

Proposed activities of this project are described on GtR as aiming to support social cohesion and to ‘work against educational segregation by describing a more favourable picture of schools that educate pupils coming from more disadvantaged socioeconomic backgrounds’ (para. 3). To do so, policy documents were analysed, and local policy‐makers and researchers were interviewed about their views on value‐added measures ‘designed to make fair comparisons between schools […] by adjusting for various background factors and prior attainment by the individual child’ (para. 1). During fieldwork in Chile, the fellow ‘worked with local policymakers, particularly at the Ministry of Education and the Quality Agency in Education supporting government's thinking around innovative school accountability and school improvement methods to account for inequality when assessing school performance’ (outcomes, para. 3). Hence, the target groups of this project, which could be the focus of psychosocial well‐being impacts, were identified by the coders as school children and their families in Chile, and potentially other LMICs in the region. During interview, the fellow reflected on more potential beneficiaries, saying, ‘because most of the schools that are described and categorised as failures, usually head teachers and the whole community, they experience a lot of mental health issues’.

The project was considered by coders to engage implicitly with the psychosocial well‐being of target groups through highlighting the contextual factors that can feed into value‐added statistics in Chilean schools as a means of reducing vulnerability, social exclusion and sense of failing associated with inequalities in the distribution of wealth. Both agreed that, had only minor changes been made to the project, there were opportunities to impact all six types of psychosocial impact (Table 6). It was also recognised that, although the main impact focus was policy, psychosocial impacts may have been explored, possibly in the data collection stage, and to have been secondary benefits which went unreported. The fellow said, ‘I realised that I have lost an opportunity for bringing well‐being’ and reflected on what would have assisted her to have incorporated psychosocial well‐being impacts more explicitly: ‘availability of data and more flexibility and overall design and longer‐term projects’.

In terms of material practices, both coders agreed that texts, including data sets, policy briefings and interviews with local policy‐makers and researchers, could have created a focus to enhance psychosocial well‐being with target groups. The fellow concurred: ‘my project wasn't thinking about well‐being or improving mental health as an impact but I could see, at the end of the project, the scope for that, especially after conducting the qualitative work’ (https://www.bristol.ac.uk/media-library/sites/policybristol/briefings-and-reports-pdfs/2017-briefings--reports-pdfs/PolicyBristol_Briefing_October_2017_Chile_Schools.pdf).

### Case 2: Creating, connecting and sustaining links with the Indonesian craft economy

Our findings demonstrate that mental health‐related grants align best with only two GCRF strategic challenge portfolios: (i) global health, as might be expected, and, more interestingly perhaps, (ii) cities and sustainable infrastructure. Case Study 2 provides an example of how mental health impact has been embedded in the latter portfolio, framed broadly in terms of economic development.

This project is described on GtR as bringing together ‘rural craft producers, fair trade businesses and design researchers in Indonesia to explore opportunities, to develop new networks, build long term partnerships and support innovation’ (para. 1). It is anticipated that ‘(t)hese initiatives will improve employment opportunities in rural areas, encourage pride in local culture, improve rural incomes and reduce pressures of migration towards cities’ (para. 3). Other producers ‘will also gain by meeting diverse craft practitioners which may stimulate thinking about new product possibilities that combine diverse skills […] building “linking” and “bridging” social capital’ (para. 8). Hence, the target groups of this project, which could be the focus of psychosocial well‐being impacts, were identified by the coders as craft producers, women and lower socio‐economic groups.

One coder noted that, although framed very much as economic, the project engaged implicitly with the psychosocial well‐being of target groups through assisting craftspeople to develop self‐esteem and ability to project oneself and one's products. The other coder drew attention to implicit psychosocial well‐being impact of valuing local identity and culture. Both agreed that, had only minor changes been made to the project, there were opportunities to promote positive sense of self, facilitate community building and support community mobilisation of target groups. To do so, one coder suggested that the researchers could have asked questions about confidence, mutual support and collaboration. The other coder suggested that benefits to well‐being through increased financial security, valuing of local identity and social cohesion could have been stated more explicitly. However, they also noted how this small grant was already achieving a lot and would likely have needed some additional resource to adequately monitor psychosocial well‐being impact, although the in‐country partner organisations may have been already observing such benefits.

In terms of material practices, both coders agreed that objects and people might have created a focus to enhance psychosocial well‐being with target groups. It was suggested that craft objects could be the focus for mutual celebration of creativity, although it was noted that these were already being used to increase sense of local pride in heritage. It was also suggested that there were massive possibilities for promoting understanding and empathy for migrant communities, and although enhanced social cohesion likely occurred, this could have been observed and discussed.

### Case 3: Building resilience and inclusion in sub‐Saharan Africa through social learning around climate risks

Our findings demonstrate that mental health‐related grants were not aligned with two GCRF strategic challenge portfolios: (i) food systems and (ii) resilience to environmental shocks and change. However, coders agreed that 50 per cent of the former and 67 per cent of the latter projects sampled within these portfolios engaged implicitly with the mental health of target groups. Case Study 3 illustrates potential for mental health impact in relation to resilience to environmental shocks and change.

This project was conducted with regard to rural communities of sub‐Saharan Africa who are vulnerable to climate‐related stressors and shocks through their dependency on agriculture and livestock. It is described on GtR as focused ‘on understanding the learning processes that build resilience and support livelihoods, which are responding to multiple pressures and opportunities across timescales in contexts that are complex and highly uncertain’ (para. 2). The target groups of this project, which could be the focus of psychosocial well‐being impacts, were identified by the coders as the vulnerable communities of the project case studies.

One coder suggested that the entire focus of the project was community resilience and, by implication, engaged multiple facets of psychosocial well‐being. Interestingly, during interview, the PI interrogated the concept of resilience stating that ‘different people have different ideas of what resilience means and resilience to me does incorporate some mental health long‐term impact’. The other coder indicated that the project engaged implicitly with the psychosocial well‐being of target groups by using a social learning approach to map the ways vulnerable communities have access to climate information and share knowledge among themselves, how outcomes highlight the relationships between power and poverty, and how poor people, particularly women, suffer exclusion. The PI also reflected on a previous project illuminating these connections and giving him pause for thought about his role as researcher: ‘the reason they weren't interested in the long‐term, the seasonal forecasts was because the women‐headed households were the last in the pecking order to be able to use the communal ploughs. So knowing what the seasonal forecast was going to be actually was depressing for them […] so I sometimes wonder that I might not even be aware of some of the impacts I have on people’.

Both coders agreed that, had only minor changes been made to the project, there were opportunities to secure all three types of social well‐being impacts, one stating it is likely that the project did facilitate community building, support community mobilisation and preserve sociocultural identity via their social networks approach, although this was not a target outcome and no data were reported.

In terms of material practices, both coders agreed that interactions between or enactments upon people might have created a focus to enhance psychosocial well‐being with target groups. Specifically identified were the workshops to map social connections. Coders suggested that participants could have been asked explicitly about the psychosocial dimensions of community resilience. This could have included articulating which social connections are important to the community, how they might build‐on and protect them, and which are difficult and might be improved. The PI also expressed awareness that during research ‘sometimes the interaction can be quite negative. I think is what I was trying to point out around the mental health impact on the people that they work with. So it's not just about listening and saying, oh I've listened to you and people feeling better for that’. The other coder suggested that participants could have been encouraged to consider how they want to build and mobilise their community and to be more inclusive. It was recognised that something similar might have been done within the project but not mentioned explicitly. This is substantiated by the PI's comment that ‘there are potentially long‐term impacts of our interactions with people and they can be positive. I'd like to hope something I've done is positive’.

## CONCLUSIONS AND RECOMMENDATIONS

This study explores if there had been opportunities, in principle, to embed psychosocial well‐being impact in research tackling global challenges across the range of SDGs, doing so through analysing grants funded by the UKRI GCRF. We provide evidence for policy‐makers, global challenge research funders and researchers that there are low hanging fruit opportunities to impact psychosocial well‐being across SDGs through routine project activities. The case is already being made strongly in relation to the development sector, but ‘(w)hile many development professionals recognise the need to do more for mental health, they do not always know where to begin’ (Ryan et al., 2019, p. 1). The same is likely true of global challenges researchers, although the urgency of doing so is heightened by reduction in the United Kingdom's ODA budget in 2021 from 0.7 to 0.5 gross national income which has had a devastating impact on GCRF funds, the projects supported and communities served.

The following recommendations are suggested by our findings. First, more work is needed to provide guidance and to learn from projects across the range of SDGs, which are already achieving mental health impact as part of their routine activities without overstretching project expertise or resource. Second, research funders could provide strategic leadership to encourage the embedding of mental health impact across the range of SDG projects, which includes working in closer partnership with researchers and other organisations in LMICs. Third, professional bodies representing academic disciplines could work with their members to identify how embedding mental health impact can facilitate achievement of their primary non‐mental health research aims and signpost resources supporting this work.

## CONFLICT OF INTERESTS

The authors are funded by the Global Challenges Research Fund (GCRF) and the pattern of projects funded by this programme constitutes a major focus of this article. The intention to conduct this work was articulated in our funding application, the GCRF has not been involved in the conduct of the research or consulted on outcomes prior to publication, and being funded by the GCRF has not knowing influenced the conduct of our study.

## ETHICS STATEMENT

This study was approved by the Ethics Committee of the School of Psychology, University of Leeds, UK, PSYC‐26, 21 April 2020.

## Supporting information


**Figure S1.** Coder agreement on opportunities for psychosocial well‐being impact
**Figure S2.** Coding by type of psychosocial well‐being impact opportunity present
**Figure S3.** Number of types of psychosocial well‐being impact opportunity agreed present per project
**Figure S4.** Coder agreement on the presence of type of material practice
**Figure S5.** Coding by type of material practice present
**Figure S6.** Number of types of material practice agreed present per project
**Figure S7.** Types of material practice as a usable focus to enhance psychosocial well‐being
**Figure S8.** Number of types of material practice as a usable focus to enhance psychosocial well‐being

## Data Availability

The data on which this article is based are available publically at https://gtr.ukri.org/ and https://public.tableau.com/app/profile/uk.research.and.innovation.ukri./viz/CompetitiveFundingDecisions2015‐16to2019‐20/UKRICompetitiveFunding.

## References

[aphw12335-bib-0001] Academy of Medical Sciences . (2020). Addressing the social determinants of global mental health in the sustainable development goals era. Workshop Report 31 October–1 November 2019. London, UK.

[aphw12335-bib-0002] Boon, R. , & Plastow, J. (Eds.) (2004). Theatre and empowerment: Community drama on the world stage. Cambridge University Press. 10.1017/CBO9780511486166

[aphw12335-bib-0003] Bruckner, T. A. , Scheffler, R. M. , Shen, G. , Yoon, J. , Chisholm, D. , Morris, J. , Fulton, B. D. , Dal Poz, M. R. , & Saxena, S. (2011). The mental health workforce gap in low‐ and middle‐income countries: A needs‐based approach. Bulletin of the World Health Organization, 89(3), 184–194. 10.2471/BLT.10.082784 21379414 PMC3044251

[aphw12335-bib-0004] Colucci, E. , & Bhui, K. (2015). Arts, media and mental health: Editorial. World Cultural Psychiatry Research Review, 10(3), 113–114. https://usercontent.one/wp/www.worldculturalpsychiatry.org/wp-content/uploads/2019/08/1-Arts-V10N3-4.pdf

[aphw12335-bib-0005] Cooke, P. , & Soria‐Dolan, I. (Eds.) (2019). Participatory arts in international development. Routledge. 10.4324/9780429399190

[aphw12335-bib-0006] De Silva, M. J. (2015). Making mental health an integral part of sustainable development: The contribution of a social determinants framework. Epidemiology and Psychiatric Sciences, 24, 100–106. 10.1017/S2045796015000049 25722030 PMC6998323

[aphw12335-bib-0007] De Silva, M. J. , & Roland, J. (2014). Mental health for sustainable development report. Global Health and Mental Health All‐Party Parliamentary Groups. https://www.mhinnovation.net/sites/default/files/downloads/resource/APPG_Mental-Health_Web.pdf

[aphw12335-bib-0008] de Witte, M. , Orkibi, H. , Zarate, R. , Karkou, V. , Sajnani, N. , Malhotra, B. , Ho, R. T. H. , Kaimal, G. , Baker, F. A. , & Koch, S. C. (2021). From therapeutic factors to mechanisms of change in the creative arts therapies: A scoping review. Frontiers in Psychology, 12. 10.3389/fpsyg.2021.678397 PMC8336579 34366998

[aphw12335-bib-0009] Duara, R. , Hugh‐Jones, S. , & Madill, A. (2018). Photo‐elicitation and time‐lining to enhance the research interview: Exploring the quarterlife crisis of young adults in India and the United Kingdom. Qualitative Research in Psychology, 1–24. 10.1080/14780887.2018.1545068

[aphw12335-bib-0010] Ehsan, A. , Klaas, H. S. , Bastianen, A. , & Spini, D. (2019). Social capital and health: A systematic review of systematic reviews. Social Science & Medicine: Population Health, 8, 100425. 10.1016/j.ssmph.2019.100425 PMC6580321 31431915

[aphw12335-bib-0011] Falkenström, F. , Ekeblad, A. , & Holmqvist, R. (2016). Improvement of the working alliance in one treatment session predicts improvement of depressive symptoms by the next session. Journal of Consulting and Clinical Psychology, 84(8), 738–751. 10.1037/ccp0000119 27213493

[aphw12335-bib-0012] Fancourt, D. , & Finn, S. (2019). What is the evidence on the role of the arts in improving health and well‐being? A scoping review. WHO Regional Office for Europe. 32091683

[aphw12335-bib-0013] Flores, E. C. , Fuhr, D. C. , Bayer, A. M. , Lescano, A. G. , Thorogood, N. , & Simms, V. (2018). Mental health impact of social capital interventions: A systematic review. Social Psychiatry and Psychiatric Epidemiology, 53(2), 107–119. 10.1007/s00127-017-1469-7 29234826 PMC6040224

[aphw12335-bib-0014] Glen, S. (2016). Inter‐rater reliability IRR: Definition, calculation. https://www.statisticshowto.com/inter-rater-reliability/

[aphw12335-bib-0015] Herrera‐Ferrá, K. (2020). Global mental health and the treatment gap: A human rights and neuroethics concern. In D. J. Stein & I. Singh (Eds.), Global mental health in practice: Global mental health and neuroethics (pp. 133–143). Academic Press. 10.1016/B978-0-12-815063-4.00009-5

[aphw12335-bib-0016] Kirschenbaum, H. , & Jourdan, A. (2005). The current status of Carl Rogers and the person‐centered approach. Psychotherapy, 42(1), 37–51. 10.1037/0033-3204.42.1.37

[aphw12335-bib-0017] Lomholt, L. H. , Andersen, D. V. , Sejrsgaard‐Jacobsen, C. , Øzdemir, C. M. , Graff, C. , Schjerning, O. , Jensen, S. E. , Straszek, S. P. V. , Licht, R. W. , Grøntved, S. , & Nielsen, R. E. (2019). Mortality rate trends in patients diagnosed with schizophrenia or bipolar disorder: A nationwide study with 20 years of follow‐up. International Journal of Bipolar Disorders, 7(1), 6. 10.1186/s40345-018-0140-x 30820700 PMC6395457

[aphw12335-bib-0029] Madill, A. , Bhola, P. , Colucci, E. , Croucher, K. , Evans, A. & Graber, R. (in submission). How can we mainstream mental health in research engaging the range of sustainable development goals? A theory of sector culture change. 10.1371/journal.pgph.0000837 PMC10022371 36962779

[aphw12335-bib-0018] Patalay, P. , Gondek, D. , Moltrecht, B. , Giese, L. , Curtin, C. , Stanković, M. , & Savka, N. (2017). Mental health provision in schools: Approaches and interventions in 10 European countries. Global Mental Health, 4, e10. 10.1017/gmh.2017.6 28596911 PMC5454766

[aphw12335-bib-0019] Patel, V. , Saxena, S. , Lund, C. , Thornicroft, G. , Baingana, F. , Bolton, P. , Chisholm, D. , Collins, P. Y. , Cooper, J. L. , Eaton, J. , Herrman, H. , Herzallah, M. M. , Huang, Y. , Jordans, M. J. D. , Kleinman, A. , Medina‐Mora, M. E. , Morgan, E. , Niaz, U. , Omigbodun, O. , … UnÜtzer, J. Ü. (2018). The *Lancet* Commission on global mental health and sustainable development. The Lancet, 392(10157), 1553–1598. 10.1016/S0140-6736(18)31612-X 30314863

[aphw12335-bib-0020] Perkins, J. M. , Subramanian, S. V. , & Christakis, N. A. (2015). Social networks and health: A systematic review of sociocentric network studies in low‐ and middle‐income countries. Social Science & Medicine, 125, 60–78. 10.1016/j.socscimed.2014.08.019 25442969 PMC5690563

[aphw12335-bib-0021] Piquette, K. E. , & Whitehouse, R. D. (Eds.) (2013). Writing as material practice. Ubiquity Press. 10.5334/bai

[aphw12335-bib-0022] Rajabzadeh, V. , Burn, E. , Sajun, S. Z. , Suzuki, M. , Bird, V. J. , & Priebe, S. (2021). Understanding global mental health: A conceptual review. British Medical Journal Global Health, 6, e004631. 10.1136/bmjgh-2020-004631 PMC7993328 33758013

[aphw12335-bib-0023] Ryan, G. , Iemmi, V. , Hanna, F. , Loryman, H. , & Eaton, J. (2019). Mental health for sustainable development: A topic guide for development professionals. K4D Emerging Issues Report. Mental Health Innovation Network and IDS. https://opendocs.ids.ac.uk/opendocs/bitstream/handle/20.500.12413/14908/K4D_MentalHealthTopicGuide_Online.pdf?sequence=2&isAllowed=y

[aphw12335-bib-0024] Ryan, G. K. , Bauer, A. , Endale, T. , Doukani, A. , Cerga‐Pashoja, A. , Brar, S. K. , Eaton, J. , & Bass, J. K. (2021). Lay‐delivered talk therapies for adults affected by humanitarian crises in low‐ and middle‐income countries. Conflict and Health, 15, 30. 10.1186/s13031-021-00363-8 33892755 PMC8062937

[aphw12335-bib-0025] Siriwardhana, C. , & Stewart, R. (2013). Forced migration and mental health: Prolonged internal displacement, return migration and resilience. International Health, 5(1), 19–23. 10.1093/inthealth/ihs014 24029841

[aphw12335-bib-0026] World Health Organization (2018). *Mental health: Strengthening our response* . https://www.who.int/news-room/fact-sheets/detail/mental-health-strengthening-our-response

[aphw12335-bib-0028] World Health Organisation (2020). *Mental health and psychosocial considerations during the COVID‐19 outbreak* . https://www.who.int/docs/default-source/coronaviruse/mental-health-considerations.pdf

