# Supplementary material for: Mainstreaming global mental health: Is there potential to embed psychosocial well‐being impact in all global challenges research?
Source: Appl Psychol Health Well Being. 2022 Jan 18;14(4):1291–313. doi: 10.1111/aphw.12335 (PMC9786259; doi:10.1111/aphw.12335)

Mainstreaming Global Mental Health: Is there Potential to Embed Psychosocial Wellbeing Impact in all Global Challenges Research?

Figure 1: Coder agreement on opportunities for psychosocial wellbeing impact

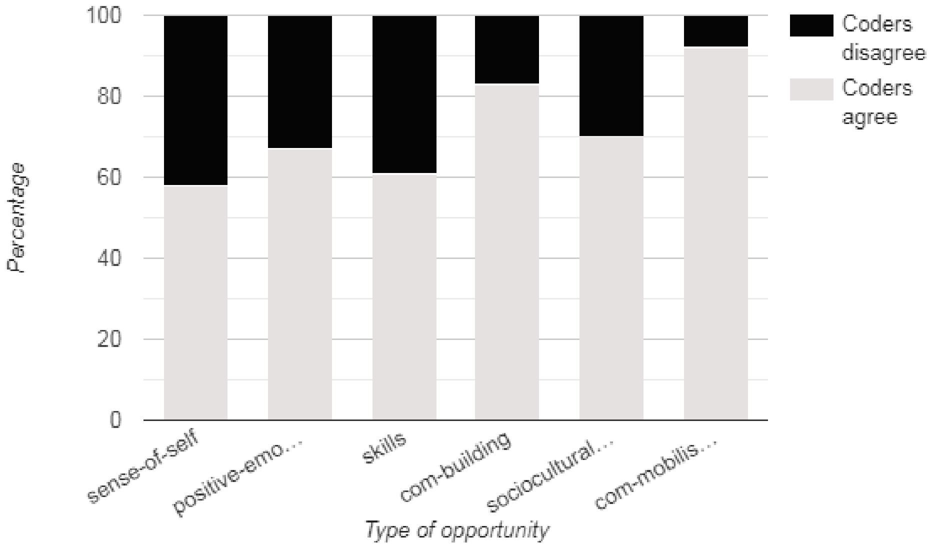

Figure 2: Coding by type of psychosocial wellbeing impact opportunity present

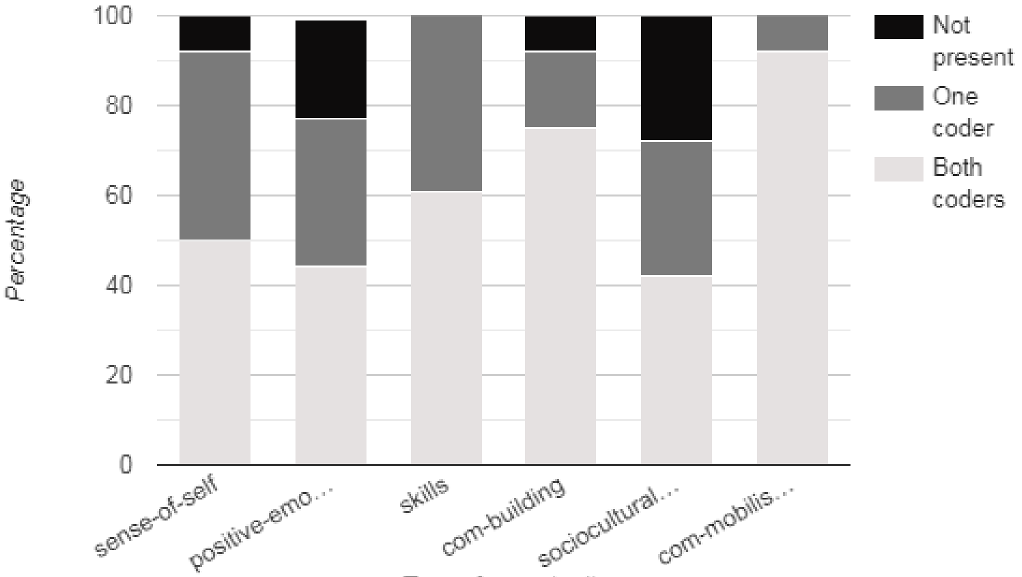

Figure 3: Number of types of psychosocial wellbeing impact opportunity agreed present per project

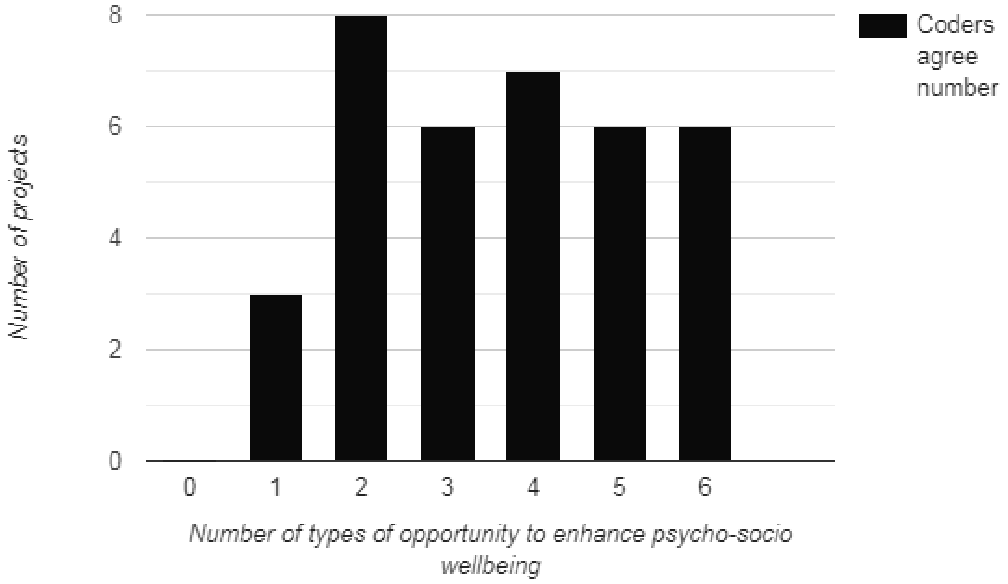

Figure 4: Coder agreement on the presence of type of material practice

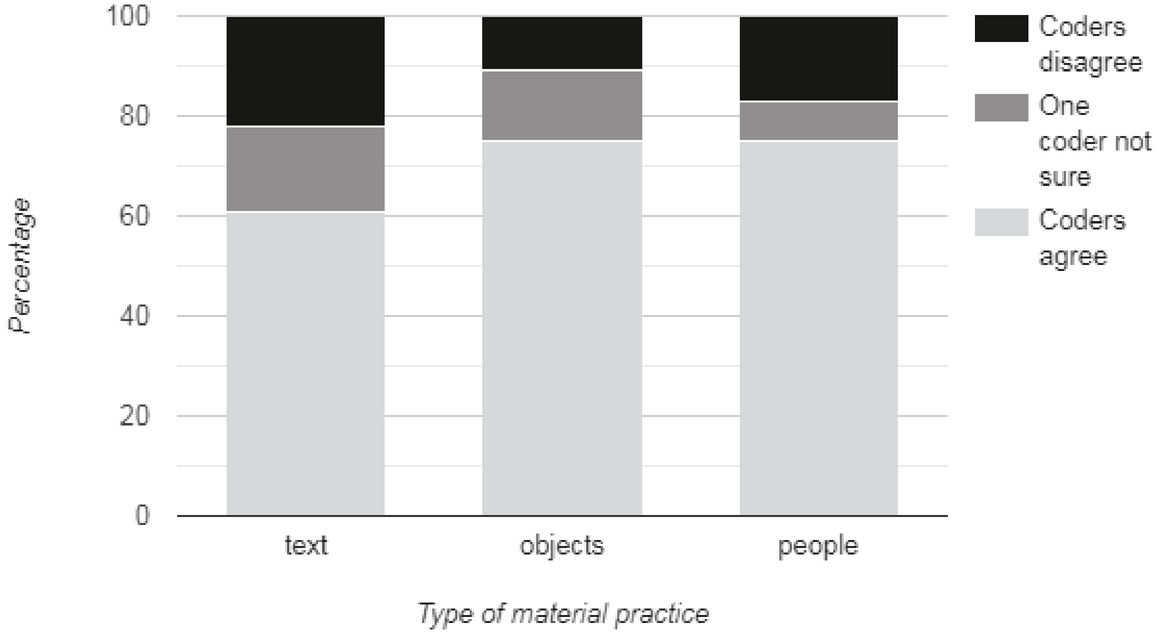

Figure 5: Coding by type of material practice present

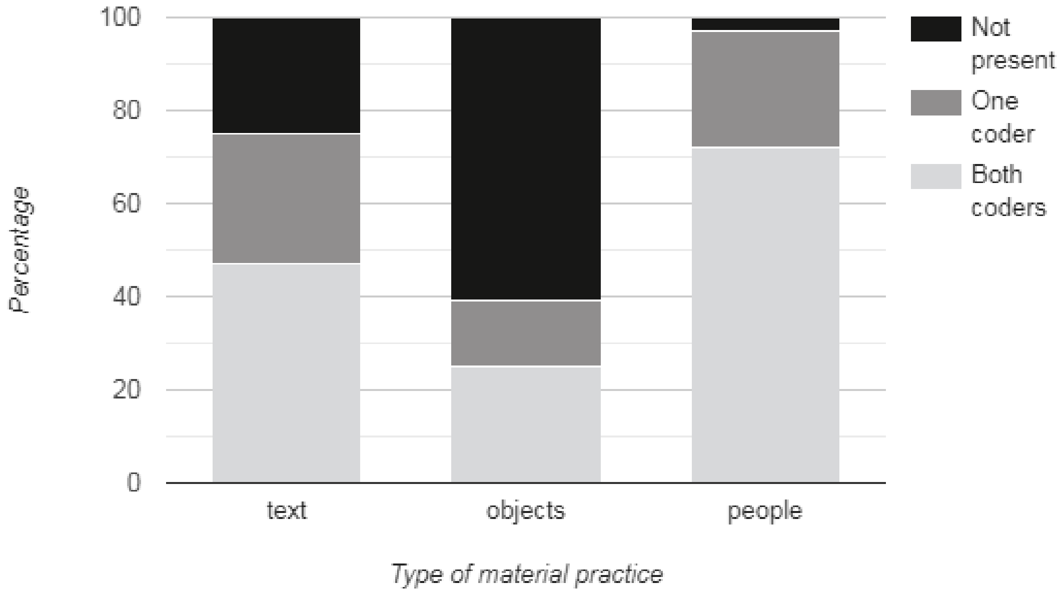

Figure 6: Number of types of material practice agreed present per project

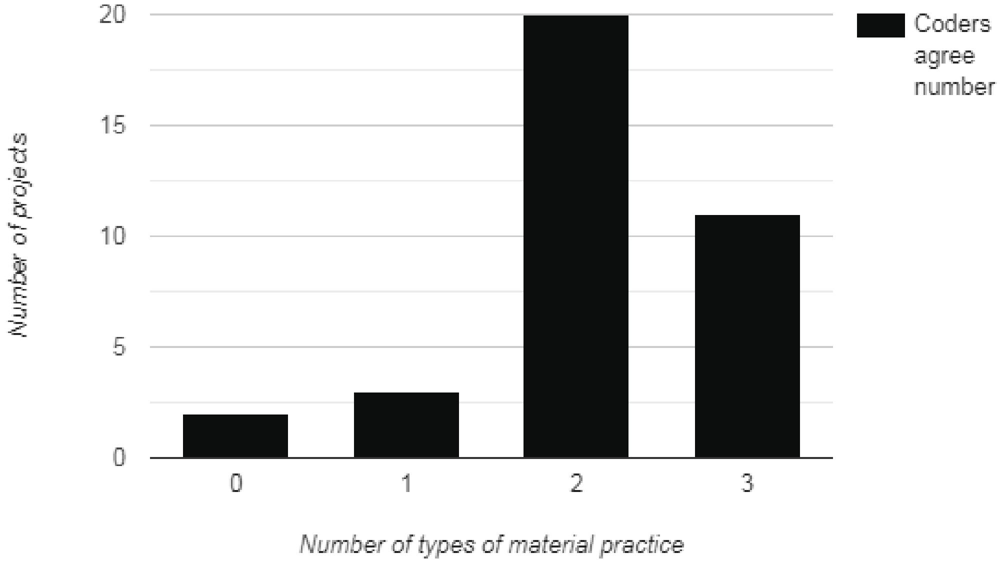

Figure 7: Types of material practice as a usable focus to enhance psychosocial wellbeing

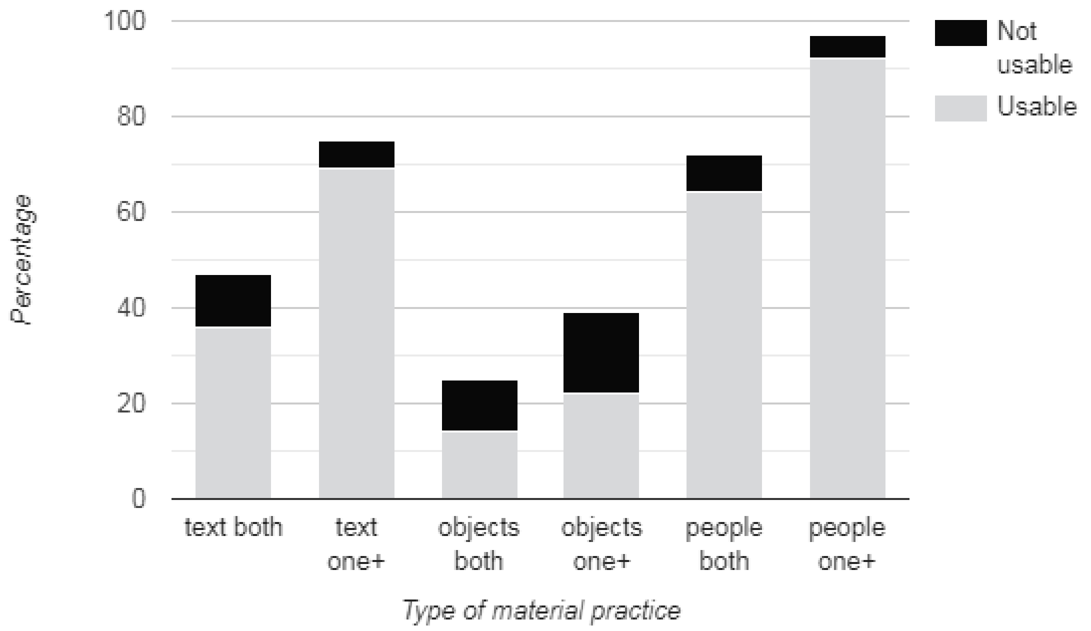

Figure 8 Number of types of material practice as a usable focus to enhance psychosocial wellbeing

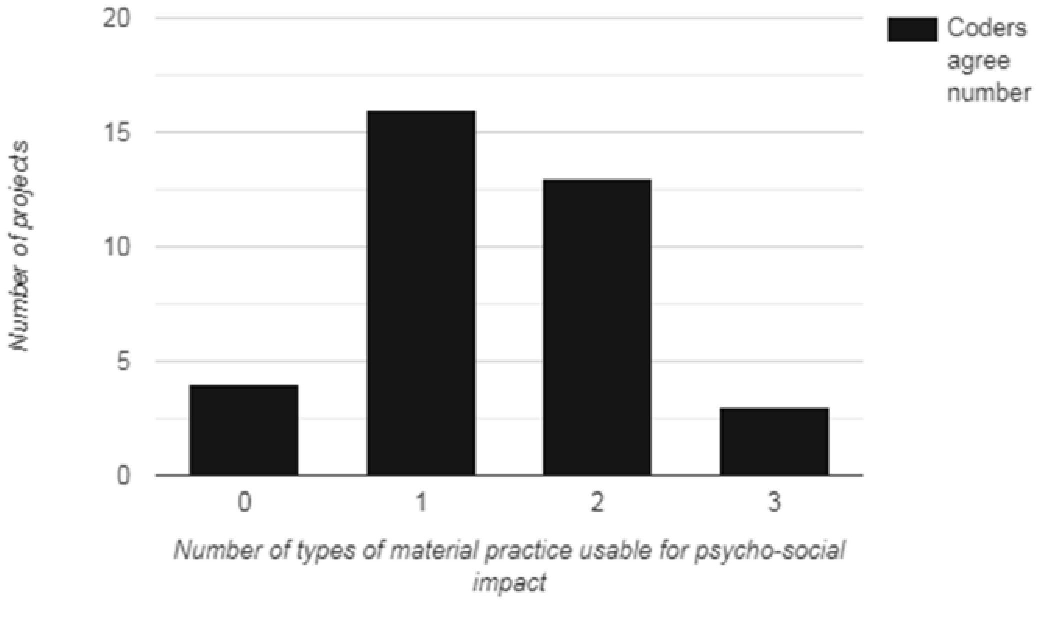

Supplement: Supplementary file 1 — Figure S1. Coder agreement on opportunities for psychosocial well‐being impact Figure S2. Coding by type of psychosocial well‐being impact opportunity present Figure S3. Number of types of psychosocial well‐being impact opportunity agreed present per project Figure S4. Coder agreement on the presence of type of material practice Figure S5. Coding by type of material practice present Figure S6. Number of types of material practice agreed present per project Figure S7. Types of material practice as a usable focus to enhance psychosocial well‐being Figure S8. Number of types of material practice as a usable focus to enhance psychosocial well‐being [file APHW-14-1291-s001.pdf]
